# Our Shared Code: The Myriad Decision and the Future of Genetic Research

**DOI:** 10.1289/ehp.121-a250

**Published:** 2013-08-01

**Authors:** Sharon Levy

**Affiliations:** **Sharon Levy**, based in Humboldt County, CA, has covered ecology, evolution, and environmental science since 1993. She is the author of *Once and Future Giants: What Ice Age Extinctions Tell Us about the Fate of Earth’s Largest Animals*.

Christopher Mason felt euphoric. On the morning of 13 June 2013 Mason, a geneticist at Weill Cornell Medical College in New York, had just heard news of the Supreme Court’s opinion in the case *Association for Molecular Pathology et al. v. Myriad Genetics, Inc., et al*. The Court had decided that “a naturally occurring DNA segment is a product of nature and not patent eligible merely because it has been isolated.”^1^

“I was ecstatic,” Mason says. “This was a huge victory for patients, scientists, and clinicians; the genome is finally free, your genes are finally yours.” For 15 years Myriad’s patents had given it a monopoly on *BRCA* gene testing in the United States, limiting the availability of the test and making it impossible for some patients to obtain a second opinion on their results. Concerned that gene patents put him and other researchers at risk of expensive lawsuits, Mason had worked as an expert witness in the case and coauthored a paper in *Genome Medicine* exploring the ways in which patents like Myriad’s clashed with basic concepts in genetics and could stifle genetic research.^2^

After the June ruling, many news outlets reported that the Supreme Court had ruled that “human genes cannot be patented.” But much of the coverage missed the ambiguity in the decision and the divide between legal doctrine and scientific understanding reflected in the case. And although the Court’s decision settled some vexing problems, many questions remain.

## Myriad’s Patent Claims

The first U.S. gene patent was granted in 1982. Since then, researchers have estimated that patents had been granted covering 20%^3^ to 41%^2^ of the human genome. And while the exact number of extant gene patents prior to the June 2013 ruling is unknown, they have been estimated to number in the thousands.^4^ However, University of Missouri–Kansas City law professor Christopher Holman says these studies overestimated the number of patent claims by counting patents that merely refer to gene sequences without asserting claims to them.^4^ (A claim is the portion of the patent that lays out exactly what the patent is intended to protect.)

The focus of the Supreme Court case was Myriad’s patents on isolated forms of the genes *BRCA1* and *BRCA2*, which its scientists had co-discovered in the early 1990s. (Researchers at the National Institutes of Health were also involved in the discovery of *BRCA1*.) These genes are associated with an increased risk of breast and ovarian cancer. Under Myriad’s patents, no other U.S. laboratory could test for these DNA sequences without risking a patent infringement lawsuit.

Myriad’s patents included some particularly far-reaching claims. Claims 5 and 6 asserted rights not only to the complete *BRCA* genes, but also to segments as short as 15 base pairs (“mers”) in length. Because nucleotide patterns repeat many times within the human genome—and, for that matter, in the genomes of other species—these claims, if enforced, would have allowed Myriad to block a wide range of research and clinical testing. Human chromosome 1, for instance, contains more than 300,000 oligonucleotides covered by the 15-mer claim on *BRCA1*.^6^

**Figure:**
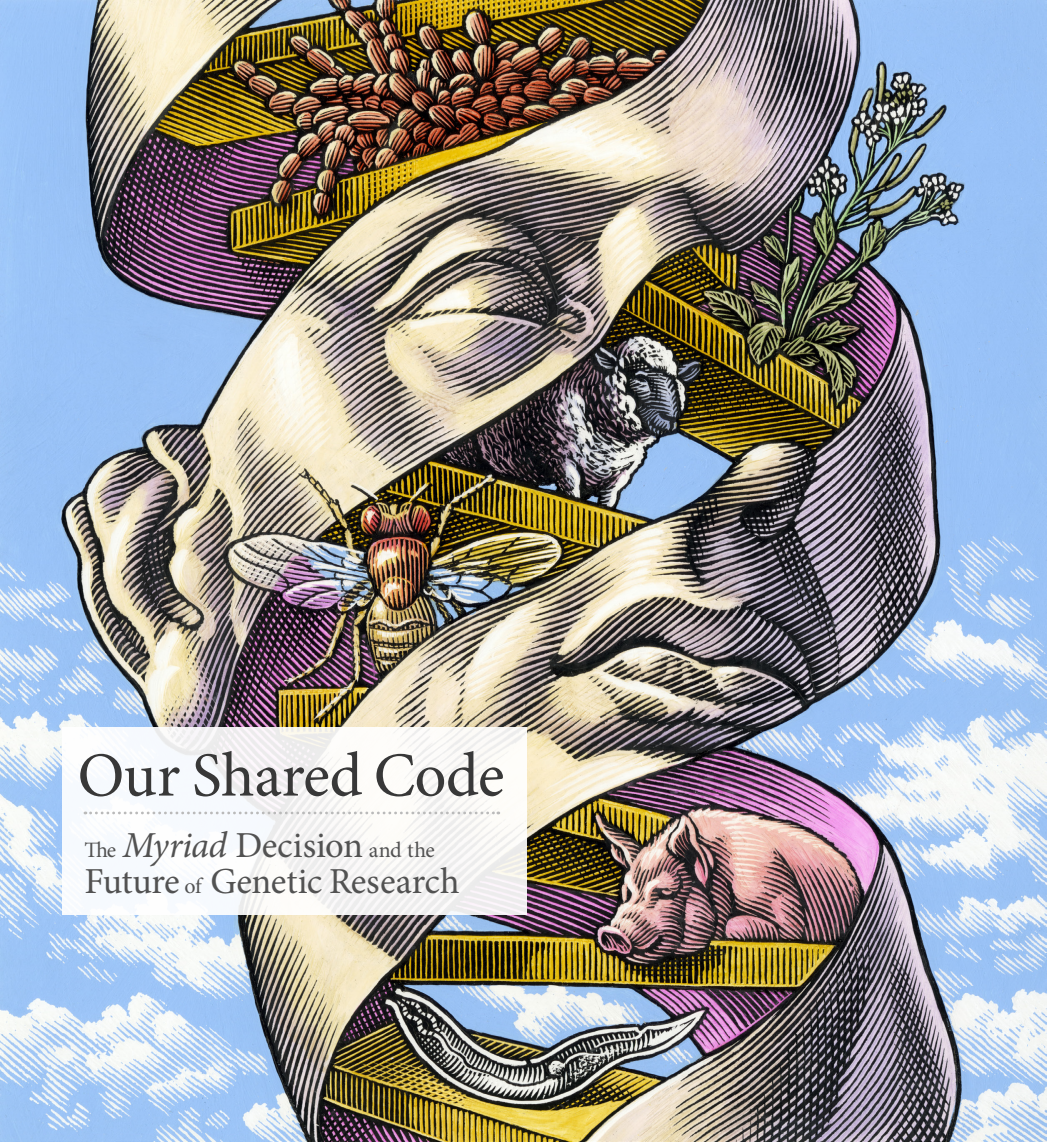
BRCA1 and BRCA2, the genes at the center of the Myriad decision, have been highly conserved throughout millions of years of evolution. The genes exist not just in humans but also many other creatures, down to invertebrates and yeasts. These tumor suppressor genes code for proteins that work to repair double-stranded breaks in DNA. Mutations of the BRCA genes can lead to improper formation of the associated proteins, which in turn may play a role in cancers of all kinds, not just breast and ovarian cancers.^14^

Because Myriad enforced its patents against competitors in clinical testing,^4^ it became the focus of a lawsuit brought by the American Civil Liberties Union (ACLU) and the Public Patent Foundation on behalf of patients, medical providers, and professional organizations, including the Association for Molecular Pathology.

In taking on the Myriad case, the Supreme Court agreed to consider the question of whether human genes could be patented—adopting the phrasing used by the ACLU in its complaint.^7^ Gregory Graff, an agricultural economist at Colorado State University, was lead author on a recent paper in *Nature Biotechnology* that warned of the potential tangle that could result from a ruling that applied only to human genes.^8^ Like Mason, he pointed out that although the Court had agreed to consider the question of whether human genes can be patented, the biological reality is that DNA is just DNA; the human genetic code is not much different from that of many other life forms.

Graff’s analysis found that valid patents claimed isolated DNA more often from plants, animals, and microbes than from the human genome. But gene sequences from many other species overlap with sections of the human genome, and given how patents are written, Graff says, the species of origin of a claimed DNA sequence is not always clear.

## Striking a Balance

The justices solved this problem by making no mention of “human” versus “nonhuman” DNA in their decision and ruled simply that naturally occurring DNA sequences are ineligible to be patented; this settles the concerns of many critics. But the Court also held that complementary DNA (cDNA) can be patented because it does not occur in nature—it is a transcript of natural protein-encoding DNA sequences from which noncoding sequences called introns have been removed. Because cDNA is synthesized and used constantly in genomic research and in pharmaceutical production, the ramifications of this decision for future studies are unclear.

“The opinion is not terribly coherent,” says Dan Burk, a professor of law at the University of California, Irvine, who holds degrees in molecular biology and biochemistry. “It’s a short opinion that leaves a lot of questions unanswered.”

The opinion, written by Justice Clarence Thomas, begins by laying out some basics of molecular biology. Thomas notes that a gene isolated in the laboratory contains the same genetic information as a gene in a living cell, and concludes that isolated genes are therefore products of nature and not patent eligible. But cDNA, which is transcribed in the laboratory from messenger RNA, is free of the introns found in the native genome. cDNA sequences are useful because they carry genetic information identical to that found in nature, but that detail was deemed irrelevant.

Burk believes this philosophical shifting of gears mid-decision shows the justices were seeking a way to limit gene patenting without undermining the numerous patents involved in the biotechnology and pharmaceutical industries. Jacob Sherkow, a fellow at Stanford University’s Center for Law and the BioSciences, agrees that the Court’s decision frees up clinical genetic testing, which nearly always uses isolated genes.

Other processes, such as splicing human DNA into bacteria in order to mass-produce a human protein, require the use of cDNA. But Sherkow believes such patents won’t present much of a practical problem. “Any clever researcher or patent agent will be able to work their way around patents on cDNAs,” he says. “Add a couple nucleotides, take out one exon, manipulate the sequence a bit, and you’re almost certain to fall outside of patent protection.”

Bioethicist David Resnik of the National Institute of Environmental Health Sciences explains that the justices were grappling with the longstanding question of just how much human ingenuity is required to transform a natural object into an invention. He thinks the Court struck a reasonable balance. “Raw sequence data will be freely available,” he says, “but significant changes to the sequence data will be protected.”

## Correcting Past Mistakes

For Sherkow, the greatest significance of the Supreme Court decision is that it ends the 30-year-old practice of granting patents on isolated DNA. However, the decision could have implications that reach far beyond gene patenting if it is used to overturn the century-old legal doctrine that allows the patenting of all sorts of biological substances isolated from nature.

That doctrine rests on a 1911 decision in *Parke-Davis v. Mulford*, a dispute over a patent on the hormone adrenaline. In his decision, Judge Learned Hand, who was then just beginning his career on the bench, allowed the patent to stand and declared that useful substances newly isolated from nature were patentable. By the end of his life, Hand would be considered an outstanding jurist, especially revered for his rulings on patents and intellectual property. In 1958 attorneys used his decision in the *Parke-Davis* case to successfully argue for their client’s right to patent vitamin B_12_.

Following the success of the claim on vitamin B_12_, Hand’s decision in *Parke-Davis* would become a touchstone for generations of patent lawyers. It was cited in the U.S. Patent and Trade Office guidelines issued in 2001.^9^ In early rounds of the *Myriad* lawsuit, the company’s attorneys cited Hand’s ruling as a crucial precedent.

That changed in 2012, after Jon Harkness, a patent attorney and a science historian at the University of Minnesota, dug through original documents in the National Archives to examine the history of the *Parke-Davis* case. He discovered that the attorneys in the case had never argued the merits of patent rights on biological substances. Learned Hand listened to a dispute over who had priority rights to the patent, not an analysis of whether molecules found in nature should be patented at all. When he stated that an isolated hormone could be patented, he was ignorant of an important Patent Office precedent established in 1889, which disallowed a patent for the fibrous core extracted from pine needles on the grounds that the claimant had invented nothing and was simply using an object that exists in nature.^10^

Harkness summed up this history in a 2011 article.^11^ “If the U.S. Supreme Court agrees to consider *Myriad*,” he wrote, “the justices should not turn to *Parke-Davis* for sage guidance from a judicial genius. Instead, they need to grapple with a difficult question that arises from this old case: Has the time come to reverse the trajectory of historical inertia that began with a small—almost inadvertent—shove in the wrong direction, a century ago, from an inexperienced and under-informed district court judge?” Soon after the article was published, Harkness notes, Myriad’s attorneys stopped mentioning *Parke-Davis* as a precedent.

Harkness now believes the Supreme Court decision in *Myriad* has corrected Learned Hand’s century-old mistake. Sherkow would welcome the demise of the *Parke-Davis* doctrine, but he isn’t sure that it’s done for; the wording of the ruling suggests, but never clearly states, that Hand’s *Parke-Davis* decision is defunct as a precedent. Just what this means in terms of future efforts to patent isolated molecules other than DNA is unclear.

“The impact of this decision could reverberate beyond genetic medicine,” says Harkness. “It might mean that chemicals found in plants or microbes—which are the sources of many pharmaceuticals—can no longer be patented.”

Resnik is concerned that doing away with patents on isolated molecules would be detrimental to the pharmaceutical, chemical, and biotechnology industries. He hopes that’s not how the Supreme Court decision will be interpreted. “Myriad’s patent claims applied to the information contained in the chemical structure of DNA, not to the exact chemical formula of the structure itself,” he says. “One could hold that you can’t patent information, i.e., sequence data, but you can patent chemical structures that you have isolated and purified.”

## Myriad’s Legacy: Proprietary Data

Myriad’s patent-authorized monopoly on *BRCA* gene testing may have ended, but its legacy will continue. Beginning in late 2004 the company chose to withhold information on variations of the *BRCA* gene from public databases. “Myriad has more data on *BRCA* mutations than anyone else,” explains Robert Cook-Deegan, a research professor in the Institute for Genome Sciences and Policy and the Sanford School of Public Policy at Duke University. He fears that proprietary databases like Myriad’s could hinder the progress of genetic medicine. “Databases and trade secrets,” he notes, “don’t expire like patents do.”

In most cases, *BRCA* analysis clearly shows whether an individual is at increased risk of breast and ovarian cancer. But some patients’ *BRCA* genes possess what are called “variants of unknown significance” (VUS). In such cases, deciding whether a patient is at elevated cancer risk is a tough call. Because it has access to information on rare *BRCA* variants in its proprietary database, Myriad claims that only 3% of its analyses are returned with a diagnosis of VUS, as opposed to about 20% for most European laboratories.^12^

Myriad has recently expanded its business into Germany, Switzerland, France, Italy, and Spain. The company claims it can offer a better standard of *BRCA* testing than any European lab thanks to the information in its proprietary database.

Because those data are held as a trade secret, Myriad’s analyses of different *BRCA* mutations have not received clinical peer review; to protect the company’s trade secret, the company expects medical providers and patients to take its conclusions on faith. In the United States, Myriad has agreements with numerous health plans that have accepted those terms. Cook-Deegan hopes European health plans and providers will push Myriad to share its data—perhaps by refusing to cover its tests until the data are made public.

Cook-Deegan acknowledges that the value of Myriad’s database will dissipate with time, as other labs compile data on *BRCA* variations. But he points out that the information should belong to the patients from whom it was gathered, not to Myriad. A group of medical professionals have launched an effort to reconstruct Myriad’s database by crowdsourcing data—having patients submit the results they obtain from Myriad to a public database.^13^

Patents are meant to serve as a bargain between inventors and the public: The workings of the invention are disclosed in the patent, and in return, the inventor gets 20 years of exclusive rights to his idea. The theory is that patent rights ultimately make scientists more willing to share their useful results. But that’s not how things have worked out in the case of Myriad and *BRCA,* according to Cook-Deegan. “Here’s a case where patents are giving rise to a huge body of trade secrets,” he notes. “The patent system is not a solution to trade secrecy in the case of genetic diagnostics. It looks like it’s the cause of the problem.”

That matters in the aftermath of the *Myriad* decision, because other nations, including Australia and members of the European Union, still allow patents on isolated DNA. Myriad is one of only three companies that refuse to share their information in public databases,^12^ but in this case, Cook-Deegan fears that the actions of a single corporation may cause a bottleneck in the progress of genetic medicine. The Supreme Court decision in the *Myriad* case is historic, but the tension between profit and scientific freedom lives on.
